# Supplementary material for: Compatibilizing Effects of Poly(lactic acid) (PLA)/Poly(vinyl butyral) (PVB)/Mica Composites
Source: Polymers (Basel). 2025 Dec 23;18(1):40. doi: 10.3390/polym18010040 (PMC12787884; doi:10.3390/polym18010040)
Supplement: Supplementary file 1 [file polymers-18-00040-s001.zip › polymers-4005633-supplementary.pdf]

Supplementary information

Compatibilizing Effects of Poly(lactic acid) (PLA)/Poly(vinyl butyral) (PVB)/Mica Composites

*Hyun-woo Lee [1], Hayeong Lee [1] and Keon-Soo Jang**

Department of Materials Science and Engineering, Division of Chemical and Materials Engineering, The University of Suwon, Hwaseong 18323, Gyeonggi-do, Republic of Korea;

* Correspondence: ksjang@suwon.ac.kr (K.-S. J.)

† These authors contributed equally to this work.

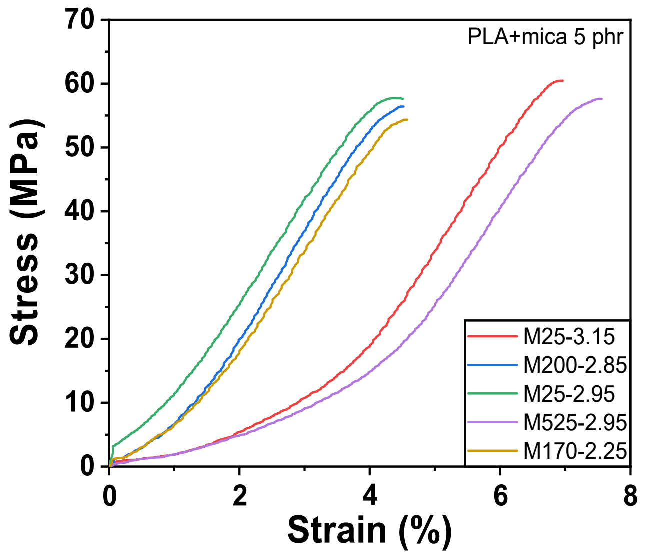

Figure S1. Stress–strain curves of PLA/mica composites as a function of particle size.

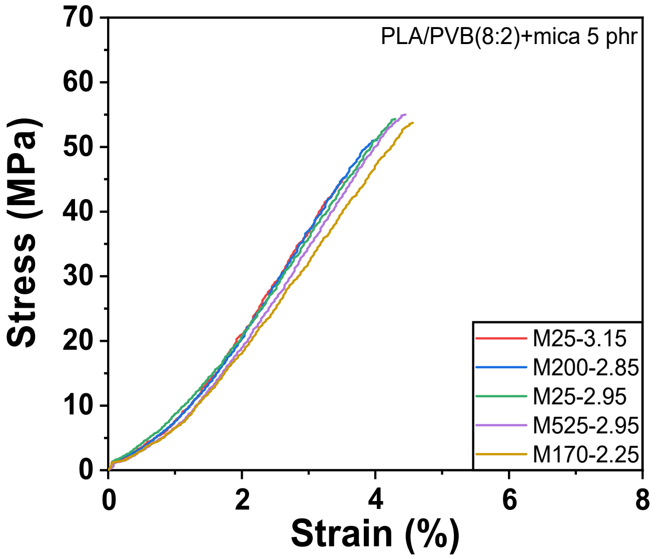

Figure S2. Stress–strain curves of PLA/PVB/mica composites as a function of particle size.
